# Clinical Characteristics and Early Diagnosis of Spontaneous Fungal Peritonitis/Fungiascites in Hospitalized Cirrhotic Patients with Ascites: A Case–Control Study

**DOI:** 10.3390/jcm12093100

**Published:** 2023-04-24

**Authors:** Yingying Jiang, Chunlei Fan, Yan Dang, Wenmin Zhao, Lingna Lv, Jinli Lou, Lei Li, Huiguo Ding

**Affiliations:** 1 Department of Hepatology and Gastroenterology, Beijing You’an Hospital, Capital Medical University, Beijing 100069, China; 2 Clinical Laboratory Center, Beijing You’an Hospital, Capital Medical University, Beijing 100069, China

**Keywords:** spontaneous fungal peritonitis, fungiascites, spontaneous bacterial peritonitis, ascites, nomogram

## Abstract

Background: Spontaneous fungal peritonitis (SFP) and fungiascites is less well-recognized and described in patients with liver cirrhosis. The aims of this study were to determine the clinical characteristics, prognosis, and risk factors of cirrhotic patients with SFP/fungiascites and to improve early differential diagnosis with spontaneous bacterial peritonitis (SBP). Methods: This was a retrospective case–control study of 54 cases of spontaneous peritonitis in cirrhotic patients (52 SFP and 2 fungiascites) with fungus-positive ascitic culture. Fifty-four SBP cirrhotic patients with bacteria-positive ascitic culture were randomly enrolled as a control group. A nomogram was developed for the early differential diagnosis of SFP and fungiascites. Results: Hospital-acquired infection was the main cause of SFP/fungiascites. Of the 54 SFP/fungiascites patients, 31 (57.41%) patients carried on with the antifungal treatment, which seemed to improve short-term (30-days) mortality but not long-term mortality. Septic shock and HCC were independent predictors of high 30-day mortality in SFP/fungiascites patients. We constructed a predictive nomogram model that included AKI/HRS, fever, (1,3)-β-D-glucan, and hospital-acquired infection markers for early differential diagnosis of SFP/fungiascites in cirrhotic patients with ascites from SBP, and the diagnostic performance was favorable, with an AUC of 0.930 (95% CI: 0.874–0.985). Conclusions: SFP/fungiascites was associated with high mortality. The nomogram established in this article is a useful tool for identifying SFP/fungiascites in SBP patients early. For patients with strongly suspected or confirmed SFP/fungiascites, timely antifungal therapy should be administered.

## 1. Introduction

Decompensated liver cirrhosis is defined as a deterioration in liver function in cirrhotic patients and is characterized by ascites, hepatic encephalopathy (HE), and portal hypertensive gastrointestinal bleeding, with an annual rate of occurrence of 5–7% [1,2]. The pathophysiological characteristics of decompensated cirrhosis include liver microcirculation disorders, local and systemic inflammation, and intestinal barrier damages, which increased the risk of infections [3]. The mortality after any infection in patients with advanced liver disease was remarkable. Arvaniti V et al. found that mortality in cirrhotic patients with infections was increased four-fold compared to those without infection [4]. Bacterial infections are common in patients with decompensated cirrhosis and can complicate the clinical course, while fungal infections are much less frequent and are associated with high fatality rates [5,6]. In a multicenter study, the 30-day mortality was 35.3% in cirrhotic patients with candidemia and intra-abdominal candidiasis [5].

Spontaneous peritonitis is one of the most common complications in patients with decompensated liver cirrhosis with ascites and is associated with mortality [7,8]. The pathomechanism, clinical features, prognosis, and therapeutic regimen of spontaneous bacterial peritonitis (SBP) is well-recognized in the literatures [9,10,11]. In contrast, spontaneous fungal peritonitis (SFP) and fungiascites remain less well known. Since signs and symptoms of SFP and fungiascites lack typical clinical features, the early differential diagnosis between SFP/fungiascites and SBP is quite difficult. The delayed management of SFP/fungiascites dramatically worsens the prognosis of patients with cirrhosis.

The aims of this study are to determine the clinical characteristics, prognosis, risk factors in cirrhotic patients with SFP/fungiascites, and to improve early differential diagnosis from SBP in clinical practice.

## 2. Materials and Methods

### 2.1. Patients

**Figure 1:**
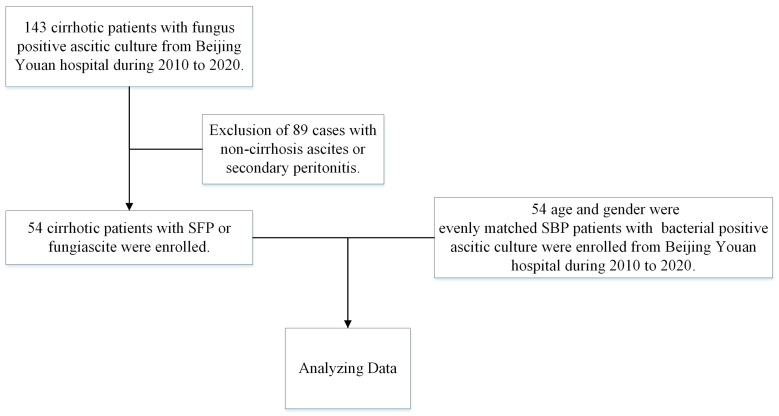
Schematic diagram of the study design.

This retrospective case–control study was conducted at Beijing You’an Hospital from 2010 to 2020. The study was conducted in accordance with the Declaration of Helsinki and approved by the Medical Ethics Review Committee of Beijing You’an Hospital, Capital Medical University (protocol code 2014L01655). A total of 54 cirrhotic patients with fungus-positive ascitic culture (including 52 SFP and 2 fungiascites) were enrolled from an SFP/fungiascites cohort. Fifty-four cirrhotic patients with bacteria-positive ascitic culture were matched with the patients in an SBP cohort by age and gender from 2010 to 2020 (Figure 1).

The inclusion criteria were as follows: (1) 18–85 years old; (2) patients diagnosed with cirrhosis and ascites, confirmed by histopathology and/or typical clinical presentation, laboratory tests, and imaging findings, with hepatocellular carcinoma (HCC) confirmed by contrast-enhanced CT/MRI imaging and/or histopathology; (3) all patients underwent cultures for both bacteria and fungus at the time of abdominal paracentesis.

The exclusion criteria were as follows: (1) non-cirrhosis ascites; (2) secondary peritonitis which was caused by gastrointestinal perforation, strangulated intestinal obstruction, or surgical operation, and so on; (3) bacteria and fungus ascites culture was not performed simultaneously at the time of abdominal paracentesis.

The primary endpoints of follow-up were (1) death or liver transplantation or (2) follow-up up to 180 days or a missed visit.

### 2.2. Ascitic Fungus and Bacteria Cultures

Ascites fluid was collected at the bedside after admission within 24 h for differential cell count and biochemical analysis and inoculated into aerobic, anaerobic, and fungal culture bottles (Becton, Dickinson and Company, East Rutherford, NJ, USA). Fungal culture was performed using Sabourand’s agar culture medium. Bacterial culture was performed by blood agar plate and MacConkey agar plate. According to colony characteristics, morphology and staining were used to determine the types of microorganisms present. The microorganism identification of ascites was performed using the Phoenix automatic identification/drug susceptibility system (Becton, Dickinson and Company, East Rutherford, NJ, USA).

### 2.3. Diagnosis of SFP/Fungiascites and SBP

Spontaneous peritonitis was defined as a peritoneal cavity infection with the absence of perforation of the intra-abdominal source or surgery. 

The diagnostic criteria of SFP are as follows: (1) fungus-positive ascitic culture regardless of bacteria-positive ascitic culture at the same time and (2) ascitic fluid polymorphonuclear leukocyte (PMN) count ≥ 250 cells/mm^3^ regardless of abdominal symptoms and sign of peritonitis or PMN count < 250 cells/mm^3^ accompanied with signs or symptoms of peritonitis, such as abdominal pain, abdominal tenderness, or rebound tenderness. The diagnostic criteria of fungiascites were fungus-positive ascitic culture regardless of bacteria-positive ascitic culture at the same time and PMN < 250 cells/mm^3^ in the ascitic fluid without signs or symptoms of peritonitis [12,13].

SBP was diagnosed according to the following diagnostic criteria: presence of bacteria-positive ascitic culture and (1) ascitic fluid PMN count ≥ 250 cells/mm^3^ regardless of abdominal symptoms or (2) PMN count < 250 cells/mm^3^ accompanied with signs or symptoms of peritonitis. 

A mixed infection was diagnosed when ascitic culture was positive for both fungus and bacteria. 

### 2.4. Clinical Data and Biochemistry

Clinical data included sex, age, laboratory test results, diagnosis, and underlying etiology from the electronic medical record. The etiologies of viral cirrhosis were hepatitis B (serum HBsAg-positive), hepatitis C (serum anti-HCV- and HCV-RNA-positive). Alcoholic, autoimmune, and metabolism-related fatty liver diseases were identified according to guidelines.

Peripheral white blood cells (WBC), neutrophilic granulocyte percentage (NEU), and platelet (PLT) counts were measured with an automated hematologic analyzer (XT-4000i, Sysmex, Kobe, Japan). An automatic biochemical analyzer (AU5400, Olympus Company, Tokyo, Japan) was used to measure biochemistry of the liver and renal system, including serum aspartate aminotransferase (AST), alanine aminotransferase (ALT), total bilirubin (TBil), albumin (ALB), and creatinine (Cr). Prothrombin time activity (PTA) was measured with an automatic coagulation analyzer (ACLTOP 700, Instrumentation Laboratory Company, Bedford, MA, USA). Serum procalcitonin (PCT) and (1,3)-β-D-glucan levels were detected by an automatic immunoanalyzer (VI-DAS, Meriere, France) and an MB-80microbial rapid dynamic detection system, respectively.

### 2.5. Statistical Analysis

Quantitative variables are presented as mean ± standard deviation (SD) or median (interquartile range). Categorical variables are presented as number (percentage). Comparisons between groups were performed using Student’s *t*-test (parametric distribution) or Mann–Whitney’s U test (nonparametric distribution) for continuous data, and chi-square test for categorical data. We performed binary logistic to determine the predictors of occurrence of SFP/fungiascites. A nomogram model was established using R software based on the results of multivariable logistic regression analysis. The nomogram provided a graphical representation of the factors that could be used to calculate the risk of SFP/fungiascites for an individual patient by the points associated with each risk factor. The predictive accuracy of the model was graphically displayed by the receiver operating characteristic curve (ROC). Cox regression analysis was used to determine the predictors of mortality. Statistical analyses for nomogram construction were performed using R software, version 4.1.3 (https://cran.r-project.org/bin/windows/base/old/4.1.3/, accessed on 17 February 2023). The GraphPad Prism software, version 6.0 (GraphPad Software Inc., San Diego, CA, USA) was used to make graphs of the comparison of mortality. All other statistical calculations were analyzed using IBM SPSS, version 22.0 (SPSS Inc., Chicago, IL, USA). A two-tailed *p* < 0.05 was considered as statistically significant.

## 3. Results

### 3.1. Clinical Characteristics of Cirrhotic Patients with SFP/Fungiascites and SBP

**Figure 2:**
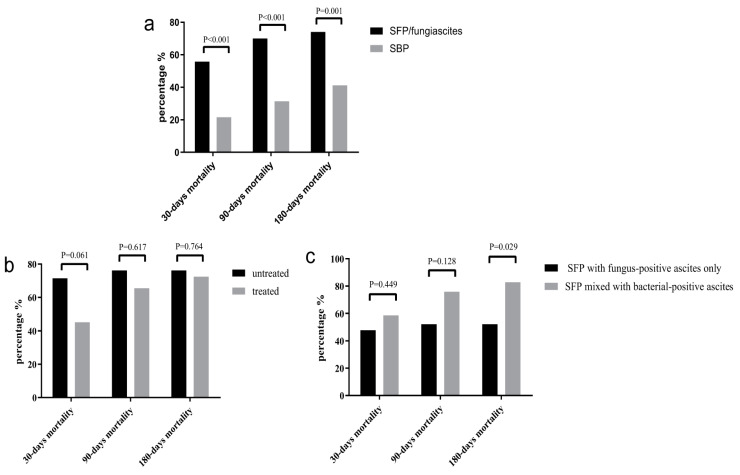
Comparison of 30-day, 90-day, and 180-day mortality. (**a**) Comparison of mortality between patients with SFP/fungiascites and SBP. (**b**) Comparison of mortality between patients treated with antifungal agents and those not. (**c**) Comparison of mortality between SFP patients with fungus-positive ascites only and SFP patients mixed with bacteria-positive ascites.

Among the 54 cirrhotic patients with fungus-positive ascitic culture in this study, the mean age at diagnosis was 58.3 years. Hepatitis virus infection was the most common etiology of cirrhosis. The time of fungus-positive culture in ascites was significantly higher than bacteria-positive culture in ascites (6 days vs. 4 days, *p* < 0.001). Hospital-acquired infection is the main cause of SFP and fungiascites infection, at a significantly higher proportion than the SBP group (83.33% vs. 29.63%, *p* < 0.001). The occurrence rates of fever, acute kidney injury/hepatorenal syndrome (AKI/HRS), and pleural effusion in SFP and fungiascites patients were far higher than in SBP patients (all *p* < 0.05, Table 1). In addition, we found that WBC, PCT, (1,3)-β-D-glucan, and model for end-stage liver disease (MELD) scores also showed higher levels in SFP/fungiascites patients. The mortality rates of patients with fungal infection in 30 days, 90 days, and 180 days were 55.78%, 70%, and 74%, respectively, which were much higher than SBP subjects (all *p* < 0.05, Figure 2a). The demographic, clinical, and laboratory characteristics of the two cohorts are summarized in Table 1. 

Among the 54 patients, 31 patients (57.41%) were treated with anti-fungal agents. Of the 23 patients who were not treated, 11 patients died and 4 patients were not scheduled to be discharged prior to positive culture; 8 patients were discharged with a better health condition. Fluconazole was the most frequently prescribed drug in the treated group. Although there was no significant difference in 30-day, 90-day, and 180-day mortality rates when comparing patients who remained on the anti-fungal treatment with those who did not, the 30-day mortality rate was much lower in patients treated with antifungal therapy than those who were not (45.16% vs. 71.43%). It seems that anti-fungal treatment could improve short-term (30-day) mortality (Figure 2b).

### 3.2. Subgroup Analyses of Fungiascites, SFP with Fungus-Positive Ascites Only, and SFP Mixed with Bacteria-Positive Ascites

Out of the 54 fungus-positive ascitic culture cirrhotic patients, 2 (3.7%) patients were fungiascites, 23 (42.6%) patients were SFP with fungus-positive ascites only, and the remaining 29 (53.7%) patients were SFP mixed with bacteria-positive ascites. Of the 29 patients with SFP mixed with bacteria-positive ascites, 18 (62.1%) had Gram-positive bacteria, 6 (20.7%) patients had Gram-negative bacteria, and 5 (17.2%) patients had mixed with Gram-positive and Gram-negative bacteria. The majority microorganism identified in Gram-positive bacteria was *Enterococcus faecium* (9/18). The most common Gram-negative bacteria was *Escherichia coli* (2/6).

There were no significant differences in transaminase, renal function, and coagulation indices between the SFP with fungus-positive ascites only group and the SFP mixed with bacteria-positive ascites group. Regarding the incidence of liver cirrhosis-related complications, there were no obvious differences in HE, AKI/HRS, and upper gastrointestinal hemorrhage between the two groups (Table 2). Although the 30-day and 90-day mortality rate showed no remarkable difference between the two groups, the 30-day and 90-day mortality in the SFP mixed with bacteria-positive ascites group were higher than that in the SFP with fungus-positive ascites only group. The 180-day mortality in SFP patients mixed with bacteria-positive ascites was significantly higher than in those with fungus-positive ascites only (*p* = 0.029) (Figure 2c).

### 3.3. Prognosis of SFP/Fungiascites

According to the univariate analysis, septic shock (hazard ratio [HR] = 3.482; 95% CI: 1.584–7.653; *p* = 0.002), HCC (HR = 2.316; 95% CI: 1.103–4.865; *p* = 0.027), PCT (HR = 1.010; 95% CI: 1.001–1.020; *p* = 0.037), NEU (HR = 1.028; 95% CI: 1.006–1.050; *p* = 0.013), and AST (HR = 1.005; 95% CI: 1.001–1.008; *p* = 0.005) were key predictors of 30-day mortality in patients with SFP/fungiascites. Furthermore, according to the multivariate Cox regression analyses, septic shock (HR = 4.171; 95% CI: 1.363–12.765; *p* = 0.012) and HCC (HR = 3.087; 95% CI: 1.196–7.973; *p* = 0.020) were independent predictors of high 30-day mortality (Table 3) in SFP/fungiascites patients.

### 3.4. Predictors for Occurrence of SFP/Fungiascites

According to the univariate analysis, AKI/HRS (odds ratio [OR] = 2.934; 95% confidence interval [CI]: 1.334–6.45; *p* = 0.007), pleural effusion (OR = 2.320; 95% CI: 1.065–5.054; *p* = 0.034), fever (OR = 3.143; 95% CI: 1.431–6.905; *p* = 0.004), high (1,3)-β-D-glucan levels (OR = 1.042; 95% CI: 1.017–1.068; *p* = 0.001), high MELD scores (OR = 1.048; 95% CI: 1.001–1.096; *p* = 0.045), and hospital-acquired infection (OR = 11.875; 95% CI: 4.715–2.911; *p* < 0.001) were predictors of SFP/fungiascites development. A multivariable analysis showed that AKI/HRS (OR = 3.568; 95% CI: 1.234–10.311; *p* = 0.019), fever (OR = 3.154; 95% CI: 1.075–9.255; *p* = 0.037), high (1,3)-β-D-glucan levels (OR = 1.029; 95% CI: 1.004–1.054; *p* = 0.022), and hospital-acquired infection (OR = 10.386; 95% CI: 3.515–30.682; *p* < 0.001) were independent predictors of SFP/fungiascites development (Table 4).

### 3.5. Development and Evaluation of a Nomogram for Early Differential Diagnosis of SFP/Fungiascites with SBP

**Figure 3:**
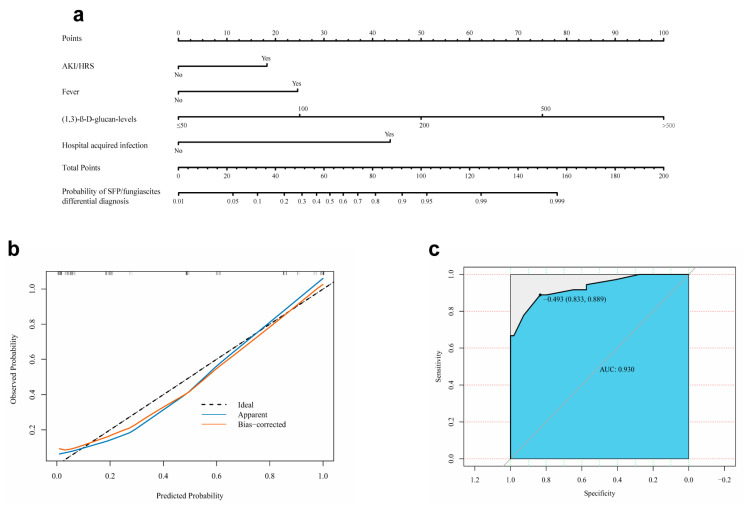
(**a**) Nomogram distinguishing SFP/fungiascites from SBP in cirrhotic patients with ascites. (**b**) The calibration plots of the nomogram. (**c**) The receiving operating characteristic (ROC) curve of the nomogram model. AUC: area under the curve.

The four independent predictors of SFP/fungiascites listed above, namely, AKI/HRS, fever, high (1,3)-β-D-glucan levels, and hospital-acquired infection, were integrated into a nomogram (Figure 3a), and the total score was calculated from the nomogram. The total points corresponded to a predicted probability of occurrence of SFP/fungiascites. The calibration plot showed good consistency between the predicted risk of SFP/fungiascites and the observed SFP/fungiascites incidence (Figure 3b). Furthermore, an ROC curve was created to estimate the predictive accuracy of the model. The area under the curve (AUC) was 0.930 (95 % CI: 0.874–0.985). When the cut-off value was −0.493, the sensitivity and specificity were 83.3% and 88.9%, respectively (Figure 3c).

## 4. Discussion

The presence of concomitant comorbidities associated with liver disease, such as obesity, alcohol consumption, malnutrition, and so on, together with dysfunction of the immune system, increased gastrointestinal permeability, and alterations in the gut microbiome, predispose cirrhosis patients to fungal infections [14,15]. A systematic review of cirrhosis and fungal infections found that abdominal infection had a mortality of about 68.3%, second only to pulmonary fungal infection. SFP/fungiascites are a devastating complication in cirrhosis with ascites, posing a great challenge for both diagnosis and therapy. To our knowledge, this is the largest study on SFP/fungiascites to date [16,17]. We described the clinical characteristics and evaluated the potential risk factors of the short-term mortality of patients with SFP/fungiascites. In addition, this study provides a nomogram model for the first time to accurately estimate the risk of SFP/fungiascites in cirrhotic patients with ascites. The nomogram model established in this paper enables physicians to distinguish SFP/fungiascites from SBP visually.

Although the incidence of SBP was much higher than that of SFP/fungiascites (7–30% vs. 1–7%), SFP/fungiascites patients exhibited significantly higher mortality rates than SBP patients [17,18,19]. Our study found that the 30-day, 90-day, and 180-day mortalities of SFP/fungiascites and SBP patients were 55.78%, 70%, and 74% vs. 21.57%, 31.37%, and 41.18%, respectively. The higher mortality in SFP/fungiascites patients can partly be attributed to the low proportion of antifungal therapy. About 42.59% (23/54) of the patients did not receive antifungal therapy in this study, which was similar to other studies (37.5–70%) [13,18,20,21]. Here, several factors should be taken into account. Firstly, SFP/fungiascites is less well recognized and lacks typical clinical signs, which delays diagnosis and the timely administration of antifungal therapy. Secondly, fungal cultures are not commonly used in clinical practice and in addition take a long time to turn positive. Thirdly, there are currently no clear guidelines of how and when to use of antifungals in SFP/fungiascites patients with ascites [7,9]. The early recognition of risk factors associated with the increased mortality of SFP/fungiascites is quite important. We found that septic shock and HCC represented independent predictors of SFP/fungiascites related to early mortality (30-day mortality). Previous studies have shown that septic shock was a predictor of mortality in both SFP patients and invasive candidiasis cirrhotic patients [22,23]. Further validation in a large-scale cohort is needed.

Traditional ascites culture usually takes 4–6 days or more to obtain the results, which may delay timely treatment for SFP/fungiascites patients. Early identification and diagnosis of cirrhotic patients with SFP/fungiascites is a critical strategy to improve the clinical prognosis of these patients. Although Huang C’s study analyzed risk factors for the development of SFP, there is no further information on how to differentiate SFP [24]. It was reported that higher MELD scores or Child–Pugh scores were associated with the development of SFP [13,25]. The results of our univariate analysis showed thar high MELD scores is a predictor of SFP/fungiascites development. In contrast, Child–Pugh scores cannot be used to predict the occurrence of SFP/fungiascites. The most probable reason is that almost all the SBP and SFP/fungiascites patients included in this study were in end-stage of liver disease (Child–Pugh C grade). Therefore, Child–Pugh scores and MELD by itself are not strongly correlated with SFP. We found that AKI/HRS, fever, high (1,3)-β-D-glucan levels, and hospital-acquired infection were independent predictors of SFP/fungiascites development. Based on the above four predictors, we developed a nomogram model to facilitate the early differential diagnosis of SFP/fungiascites. The nomogram performed well in predicting SFP/fungiascites development with an AUC of 0.930. The four predictive variables can be readily ascertained, making the use of the nomogram feasible in clinical practice.

It is easy to understand that hospital-acquired infection, fever, and high (1,3)-β-D-glucan levels were predictors of SFP/fungiascites development. It was found that hospital-acquired infection is the main route of SFP/fungiascites. Elevated serum (1,3)-β-D-glucan levels are generally common in patients with invasive fungal infections, and (1,3)-β-D-glucan tests are also conventional in clinical practice. Once fungal infection is suspected clinically, a (1,3)-β-D-glucan test should be performed promptly to avoid delayed diagnosis. We hope that this study can increase doctors’ awareness of fungal infections, which would be conducive to the early diagnosis of SFP/fungiascites using (1,3)-β-D-glucan test. As for AKI/HRS, previous studies have shown that AKI was a predictor of fungal infection development in patients with or without cirrhosis [26,27]. Moreover, it was revealed that AKI was associated with exaggerated immune response. Evidence from animal studies has shown that immune response was impaired in animals with AKI and thus developed more serious infection [26,28]. The mechanism of the association between AKI/HRS and SFP/fungiascites needs further investigation.

In the literature, approximately 60% of bacterial infections in cirrhotic patients are community acquired, and the causative organisms are Gram-negative bacteria, especially Escherichia coli [29,30]. The pattern of hospital-acquired bacterial infection is different: about 60% of these patients are infected with Gram-positive cocci, which are more resistant to antibiotics than those found in community-acquired infections [31,32]. It has been reported in the literature that the 30-day survival rate of hospital-acquired SBP infection is about 20%, which is far less than community-acquired SBP [10,33]. The clinical characteristics of SFP patients mixed with bacteria-positive ascites were analyzed in this paper. Our study found that the proportion of Gram-positive bacterial infection in SFP patients mixed with bacteria-positive ascites was 62.1% (18/29). Since most of the patients with fungal infections in our study had hospital-acquired infections, it is easy to understand that Gram-positive bacteria were the main organisms causing infections, and this was consistent with the current bacterial distribution characteristics of nosocomial infections in cirrhotic patients. We found that the mortality rate in the mixed fungal and bacterial infection group was obviously higher than SFP patients with fungus-positive ascites only and SBP patients. Appropriate selection of therapy can contribute to improved clinical outcomes. Thus, it is essential for physicians to determine how to efficiently prescribe antibacterial and antifungal drugs.

Though there are no guidelines recommending how and when to use of antifungals in SFP/fungiascites patients with ascites, previous studies have made helpful comments on the management of SFP [17,20,22]. All cirrhotic patients with ascites should undergo diagnostic puncture immediately after admission. Given the low incidence of SFP/fungiascites, prophylaxis is not necessary. When symptoms of spontaneous peritonitis do not improve after 48–72 h of empiric antibacterial therapy in patients with high risk for SFP, an antifungal should be added empirically. Echinocandins should be considered as empirical antifungal therapy for patients with suspected SFP. Echinocandins are also recommended for patients with hospital-acquired SFP and community-acquired SFP. When sensitivity tests are available, a reduction to fluconazole is appropriate, which could reduce the emergence of resistant microorganisms. In patients with mixed fungal and bacterial infection, third-generation cephalosporins may not be the first choice of antibiotics when Gram-positive bacteria were predominant [34,35]. Effective use of broad-spectrum antibiotics empirically and subsequent targeted antibiotic therapy according to drugs sensitivity testing is essential.

Lastly, our study has some limitations. This retrospective cohort study included a relatively small sample size. Secondly, the low prevalence of SFP/fungiascites makes it difficult to perform internal validation. Furthermore, validation using a multicenter external cohort will be needed in the future.

In conclusion, SFP/fungiascites is a severe complication of decompensated cirrhosis associated with high mortality. The nomogram established in this article provides a useful tool for distinguishing SFP/fungiascites from SBP patients early. For patients with strongly suspected or confirmed SFP/fungiascites, timely antifungal therapy should be administered to improve patient outcomes.

## Figures and Tables

**Table 1:** Baseline clinical characteristics of the SFP and SBP cohort.

Variable	SBP (n = 54)	SFP ^a^/Fungiascites (n = 54)	*p*-Value
Ages (years)	58.72 ± 11.72	58.30 ± 10.62	0.843
Male n (%)	40 (74.07%)	39 (72.22%)	0.828
Antifungal therapy n (%)	-	31 (57.41%)	-
Etiology			
Viral n (%)	22 (40.74%)	27 (50.00%)	0.334
Alcoholic n (%)	12 (22.22%)	12 (22.22%)	1
Others n (%)	20 (37.04%)	15 (27.78%)	0.304
Fungus			
Candida albicans n (%)	-	23 (42.59%)	-
Candida glabrata n (%)	-	10 (18.52%)	-
Candida tropicalis n (%)	-	9 (16.67%)	-
Candida parapsilosis n (%)	-	6 (11.11%)	-
Others n (%)	-	6 (11.11%)	-
Time of ascites submitted for examination (day)	7.5 (0, 16)	6.5 (1, 18.25)	0.599
Time of ascites culture positive (day)	4 (3,4)	6 (5,7)	<0.001
Hospital-acquired infection n (%) ^b^	16 (29.63%)	45 (83.33%)	<0.001
Fatigue n (%)	53 (98.15%)	54 (100%)	1
Fever n (%)	18 (33.33%)	33 (61.11%)	0.004
Abdominal swelling n (%)	52 (96.3%)	53 (98.15%)	1
Abdominal tenderness n (%)	52 (96.3%)	39 (72.2%)	0.002
Abdominal rebound tenderness n (%)	30 (55.6%)	22 (40.7%)	0.123
Malnutrition n (%)	34 (62.96%)	38 (70.37%)	0.414
HCC n (%)	16 (29.63%)	23 (42.59%)	0.161
HE n (%)	16 (29.63%)	18 (33.33%)	0.727
Variceal bleeding n (%)	10 (18.52%)	18 (33.33%)	0.079
AKI/HRS n (%)	17 (31.48%)	31 (57.41%)	0.007
Pleural effusion n (%)	18 (33.33%)	29 (53.7%)	0.033
Septic shock n (%)	6 (11.11%)	12 (22.22%)	0.121
Diabetes n (%)	15 (27.78%)	17 (31.48%)	0.673
Hypertension n (%)	10 (18.52%)	13 (24.07%)	0.481
SAAG, g/L	21.13 ± 4.91	20.26 ± 7.17	0.349
WBC, 10^9^/L	5.23 (3.25,7.42)	7.75 (3.81,13.52)	0.016
NEU, %	73.49 ± 15.47	77.96 ± 13.13	0.108
PLT, 10^9^/L	77 (45.75,120.5)	74.5 (44.25,101)	0.958
ALT, U/L	21.5 (13,42.5)	18.4 (11.83,37.83)	0.408
AST, U/L	50.5 (25,88)	44.6 (27.63,68.7)	0.669
TBIL, μmol/L	54.05 (27.28,134.38)	59.55(39.95,121.58)	0.432
ALB, g/L	28.32 ± 4.86	29.24 ± 3.87	0.281
PTA, %	55.52 ± 22.62	52.76 ± 20.98	0.512
Cr, μmol/L	84.5 (62.25,124.5)	107 (70.6,155)	0.050
PCT, ng/mL	0.28 (0.15,2.48)	1.71 (0.25,5.51)	0.026
CRP, mg/L	17.55 (12.83,53.93)	51.5 (19.45,96.03)	0.126
(1,3)-β-D-glucan levels, pg/mL	10 (10,10)	53.55 (11.53,249.75)	<0.001
Child-Pugh	10.59 ± 1.84	11.09 ± 1.65	0.140
MELD	14.65 ± 8.00	18.19 ± 9.56	0.041

^a^ the SFP includes SFP with fungus-positive ascites only and SFP mixed with bacteria-positive ascites. ^b^ Hospital-acquired infection is defined as a case of infection acquired in the hospital, occurring 48–72 h after hospital. All data are presented as n (%), mean ± SD or median (interquartile range). Abbreviations: SD: standard deviation; HE: hepatic encephalopathy; AKI: acute kidney injury; HRS: hepatorenal syndrome; SAAG: serum ascites albumin gradient; WBC: white blood cells; NEU: neutrophilic granulocyte percentage; PLT: platelets; ALT: alanine aminotransferase; AST: aspartate transaminase; TBIL: total bilirubin; ALB: albumin; PTA: prothrombin time activity; Cr: creatinine; PCT: procalcitonin; CRP: C-reactive protein; MELD: model for end-stage liver disease.

**Table 2:** Comparison of fungiascites, SFP with fungus-positive ascites only, and SFP mixed with bacteria-positive ascites.

Variable	Fungiascites (n = 2)	SFP with Fungus-Positive Ascites Only (n = 23)	SFP Mixed with Bacteria-Positive Ascites (n = 29)	*p*-Value ^a^
HE n (%)	0 (0%)	6 (26.08%)	12 (41.38%)	0.250
Variceal bleeding n (%)	0 (0%)	8 (34.78%)	10 (34.48%)	0.982
AKI/HRS n (%)	0 (0%)	15 (65.22%)	16 (55.17%)	0.463
Pleural effusion n (%)	1 (50%)	13 (56.52%)	15 (51.72%)	0.730
Septic shock n (%)	1 (50%)	4 (17.39%)	7 (24.14%)	0.803
WBC, 10^9^/L	12.84	8.58 (3.46,16.03)	7.62 (4.75,11.56)	0.625
NEU, %	78.55	78.05 ± 13.28	77.86 ± 13.32	0.960
PLT, 10^9^/L	102	72 (54,96)	78 (31,119)	0.706
ALT, U/L	7.6	28 (15,40.6)	16.3 (7.5,31.15)	0.068
AST, U/L	13.65	51.3 (30,74.7)	42 (21,80)	0.269
TBIL, μmol/L	62.15	65.4 (42.8,162.4)	52.2 (39.9,86.6)	0.507
ALB, g/L	32.7	30.33 ± 3.46	28.13 ± 3.69	0.033
PTA, %	77.5	52.26 ± 23.69	51.45 ± 15.14	0.881
Cr, μmol/L	315.25	126.16 (77,145)	100.5 (66.25,155)	0.478
PCT, ng/mL	0.9	2.12 (0.26,5.93)	1.58 (0.2,4.93)	0.465
CRP, mg/L	26.1	41.5 (17.6,90.1)	71.65 (24.35,150.5)	0.322
(1,3)-β-D-glucan levels, pg/mL	143.19	53.05 (10,431.93)	50.4 (13.65,131.55)	0.829
Child–Pugh	10	10.74 ± 1.60	11.45 ± 1.66	0.127
MELD	11.08	18.56 ± 9.19	18.54 ± 10.17	0.995

Notes: Since there are only two cases of data in fungiascites group, the quantitative data of this group took the average. ^a^ Comparison between SFP only and SFP mixed with bacteria-positive ascites.

**Table 3:** The risk factors associated with 30-day mortality in patients with SFP/fungiascites.

Variable	HR	95%CI	*p* Value
Univariate analysis			
Septic shock	3.482	1.584–7.653	0.002
HCC	2.316	1.103–4.865	0.027
PCT	1.010	1.001–1.020	0.037
NEU	1.045	1.010–1.080	0.010
AST	1.005	1.001–1.008	0.005
Multivariate analysis			
Septic shock	4.171	1.363–12.765	0.012
HCC	3.087	1.196–7.973	0.020

**Table 4:** Risk factors for SFP/fungiascites occurrence in cirrhotic patients with ascites.

Variable	OR	95% CI	*p* Value
Univariate analysis			
AKI/HRS	2.934	1.334–6.45	0.007
Pleural effusion	2.320	1.065–5.054	0.034
Fever	3.143	1.431–6.905	0.004
(1,3)-β-D-glucan levels	1.042	1.017–1.068	0.001
MELD	1.048	1.001–1.096	0.045
Hospital-acquired infection	11.875	4.715–2.911	<0.001
Multivariate analysis			
AKI/HRS	3.568	1.234–10.311	0.019
Fever	3.154	1.075–9.255	0.037
(1,3)-β-D-glucan levels	1.029	1.004–1.054	0.022
Hospital-acquired infection	10.386	3.515–30.682	<0.001

## Data Availability

Data presented in this study are available from the corresponding author upon request.
